# Cognitive Enhancement through Differential Rope Skipping after Math Lesson

**DOI:** 10.3390/ijerph20010205

**Published:** 2022-12-23

**Authors:** Johannes Burdack, Wolfgang I. Schöllhorn

**Affiliations:** Department of Training and Movement Science, Institute of Sport Science, Johannes Gutenberg-University Mainz, 55128 Mainz, Germany

**Keywords:** differential learning, arithmetic, math, cognitive learning, physical activity, adolescents, rope skipping

## Abstract

Numerous studies have shown cognitive enhancement through sport and physical exercise. Despite the variety of studies, the extent to which physical activity before or after a cognitive learning session leads to more effective cognitive enhancement remains largely unresolved. Moreover, little attention has been paid to the dependence of the motor learning approach then applied. In this study, we compare the influence of differential with uniformly rope skipping directly succeeding an acquisition phase in arithmetic mathematics. For three weeks 26 pupils, 14 female, 12 male, and 13.9 ± 0.7 years old, completed nine 15 min exercises in arithmetic math, each followed by 3 min rope skipping with heart rate measurement. Arithmetic performance was tested in a pre-, post- and retention test design. The results showed a statistically significant difference between the differential and the control groups within the development of arithmetic performance, especially in the retention test. There was no statistical difference in heart rate. It is suggested that the results provide evidence for sustainable improvements of cognitive learning performance by means of highly variable rope skipping.

## 1. Introduction

Sustainability has gained more and more social importance in recent times. This development is also evident in learning research, where cognitive enhancement is no longer only intended to be achieved on short-term successes until the next performance review, but on the subsequent process of sustainable learning. However, practitioners still frequently report a conflict of objectives between sustainable learning and efficient acquisition, as sustainable learning is often associated with a significantly higher time commitment. To achieve time-limited yet efficient acquisition, a variety of approaches has been proposed in recent decades. These range from physical strategies, such as electrical or magnetic stimulation, to biochemical strategies, such as nutrition or recreational drugs, to behavioral strategies [1].

One of the behavioral strategies is to use physical exercise to enhance cognitive learning. Numerous studies with various sport and movement approaches indicate not only a positive impact of sport or everyday movements on acute and sustainable cognitive performance but also on cognitive learning.

At the phenomenological level, two main categories of operationalization of this goal can be distinguished, within which further subcategories can be differentiated.

(1) The first main category relates to the content of intervention and testing that includes (a) the type and intensity of the intervention, (b) the diversity of exercises during intervention, and (c) the type of test.
  (a) The first subcategory refers to type and intensity of the physical activity performed during the intervention. Here, typically endurance related sports form one group [2,3,4,5,6,7,8,9], while another group is based on more coordinative and cognitive demands [3,10,11,12,13,14,15], and the third group can be considered more strength-oriented activities [10,16,17,18]. Regarding type and intensity, we observed that especially endurance-related movements under relatively high intensity [19] as well as coordinative movements had a supportive influence on learning, whereas strength-oriented movements did not achieve statistical differences.
  (b) The second subcategory can be assigned to studies on the effect of exercise sequence and diversity therein during intervention. Here, a basic distinction can be made between a constant and a variable exercise schedule. Within a variable exercise schedule, an additional distinction is observable between blocked, serial, and randomized practice sequence. It was shown that more variable schedules with serial and randomized order of discretely described skills, according to the contextual interference theory [20], or one or more skills including additional movement variation, according to the differential learning (DL) theory [21,22], lead to increased learning progress in motor learning and also generate brain states that are positively associated with cognitive learning [23,24,25]. In this context, comparisons of the contextual interference and DL approaches in EEG studies in badminton [26,27,28] and soccer [29] showed brain states more conducive to learning in favor of DL. More variation in motor activity consequently seems to have a more positive effect on cognitive learning, although too much variation can also cause detrimental stress [11,30], which could have an inhibitory effect.
  (c) The third subcategory refers to the type of cognitive performance that was used to test the effects. We can distinguish between specific cognitive tasks, such as learning a language or mathematics, and non-specific cognitive constructs, such as working memory and/or executive functions. In both areas, physical activity was associated with positive effects on learning [3,7,8,9,10,16,31,32].
(2) The second main category relates to aspects of time that are related to (a) the duration and frequency of intervention and to (b) the relative moment of intervention.
  (a) This subcategory refers to the dependence of the effects on the overall duration and frequency of the physical activity intervention. Here, acute, medium, and long-term effects of physical activity on cognitive performance are to be distinguished [33,34]. Acute effects are related to the consequences immediately after sporting activities [4,5,6,13,15,17,18,35,36,37,38]. The medium and long-term effects refer to the effects after repeated exercise, dependent on the frequency of application. Medium-term effects are assigned to interventions lasting up to a time scale of several weeks [13,31], while long-term effects are assigned to the practice of sport over several years on an even longer timescale [39,40,41,42,43,44].
  (b) This subcategory refers to the extent to which motoric/sport activities are applied before or after the object to be learned (cf. category 1b). In the field of cognitive learning research, these phenomena are referred to as pro- and retroactive interference (earlier also called inhibition). Proactive interference refers to the detrimental influence or superimposition of newly acquired memory content on previously learned content. This is typically associated with increased activity in the higher frequency bands in the prefrontal cortex [45]. Analogously, retroactive interference refers to the unfavorable effects of new learning on the recall of previously learned material and is mainly associated with hippocampal activity [46,47,48,49,50,51]. When comparing the effects of pro- and retroactive interference, it is perceived that retroactive interference could have a stronger effect on memory formation [52]. Contrary to inhibitory interference effects, findings from the field of meditation and eastern martial arts provide evidence that pre- and post-active meditation can have positive effects on memory consolidation [53,54,55]. In addition, more passive approaches such as sleeping or napping after an activity also indicate positive effects on just that [56,57,58,59].

As shown in Table 1, in the studies investigating a direct temporal relationship between exercise and cognition, it is apparent that the relative timing of athletic or motor exercise always preceded the investigation of cognitive performance. This usually had the goal of creating a good learning environment in terms of better oxygenation or beneficial brain states. In difference to the classical understanding of cause and effect, sustainable learning presupposes the process of memorization that follows the execution of the learning content.

For the explanation of the phenomena that are related to the effects of sports on cognition, two main physiologic mechanisms are pursued. One relates to (i) metabolism and is based on a rather indirect mechanism via respiratory and cardiac parameters. The second is more oriented on direct (ii) neurophysiological mechanisms.

(i) With regard to the metabolic effects of sport, increased cortical blood flow after physical activity has been shown [60]. This increase in blood flow is associated with a positive influence on the oxygen and glucose supply to the brain and stimulation of the metabolism [61]. The increased metabolism is somewhat vaguely assumed to be responsible for improved cognitive processes [62]. Furthermore, with regard to the neurochemical milieu, a direct influence on the chemical processes in nervous tissue is assumed by sports [61,63]. Neurotransmitters enable the transfer of stimuli between nearby cells through physical activity. An increased release of neurotrophins was found, which are supposed to stimulate the growth of nerve cells and the formation of synapses [64]. In addition, aerobic exercise has been associated with an increase in BDNF levels [65,66], which could have a positive effect on learning as well [67].
(ii) On the neurophysiological level, the successful learning of motor and cognitive skills is found to be accompanied by activation patterns of the brain, measured by fMRI or EEG, that can be influenced by physical movements. According to the hypofrontality-hypothesis [34,68] changes of the cortical activity in frontal brain areas in terms of increased alpha and theta together with decreased beta frequency bands lead to an increased probability of improved cognitive performance. Corresponding changes in brain activity could be achieved for example by continuous cardiovascular training of 80% intensity with a duration of about 20–40 min [2,4,5]. A similar effect on brain activity is seen by two other movement characteristics. One is by coordinatively demanding movements [11,26,27,28,29,30], and the other is by switching randomly between selected movements [11,23,24,26,27,28,29,30]. Alternatively, the sequence of exercises as they are discussed in motor learning approaches are associated with having influence on the brain activity. A review [23] related to the contextual interference based learning approach in fine motor movements [20] showed greater cognitive engagements with randomized exercise order compared to blocked order. However, it needs to be considered that all studies concerned only movements with small number of degrees of freedom with dominant visual influence and thus a transfer to movements with large number of degrees of freedom, like in most sports, is not readily possible [25]. Additionally, using the DL approach [21,22], only 15 min of training in badminton serve [26,27,28] and soccer goal kick [29] showed increased alpha and theta activity in the prefrontal cortex compared to repetitive learning [69]. Rope skipping studies on the DL approach with only single three minutes bouts also found that the frequency with which new tasks were announced had a significant impact on brain activity. In this regard, too high a frequency (one task every 1 to 2 s) appeared to produce too much mental stress [11], whereas a frequency of one task every 10 to 20 s provided particularly positive indications [30].

Putting all together, two aspects stand out that previous studies have neglected so far. The first of these concerns category 2b on the chronological order of exercise and learning: the influence of physical exercise following a cognitive learning unit has not yet been studied. This is somehow surprising, since active approaches like retroactive interference [49,50,51] and meditation [53] or more passive approaches like napping and sleeping [56,57,58,59] have already shown that an intervention subsequent to learning can positively influence learning.

Related to the above category 1b on the type of sporting activity, the evidence from movement research related to brain activity provides further approaches to differentiation. As shown in (ii), differences between different motor learning approaches and the exercise sequence have been demonstrated in the context of different cortical activation. However, the extent to which this specifically affects cognitive learning has not yet been investigated.

To link previous findings to the research gaps we found, in this study we examine the midterm impact of physical activity conducted under the repetitive and DL approach, immediately after cognitive acquisition learning, on performance in cognitive learning. Specifically, we choose rope skipping as a gross motor movement, coordinatively demanding as well as endurance stimulating exercise as a physical activity to be performed after learning math skills. The two learned math skills were mental arithmetic and fractions. In terms of engaged cognitive functions, mental arithmetic appears to be particularly related to visuo-spatial working memory and mental rotation [70], and fractional arithmetic appears to require inhibitory control to suppress previously learned experience (e.g., overcoming the integer bias; $\frac{2}{3}>\frac{4}{7}$) [71]. Overall, a strong relationship between executive functions, especially working memory, and mathematics achievement in children has been demonstrated [72].

## 2. Material and Methods

### 2.1. Participants

Twenty-six healthy students (13.9 ± 0.7 years) participated in this study. All students were novices in rope skipping but engaged in moderate sports (<5 h) per week; only two performed competitive sports (8–12 h a week). According to the pretest ranks, all subjects were parallelized into two groups, a test and a control group. The test group performed a rope skipping intervention according to the principles of DL [21,22] and the control group according to repetitive learning [69] (more detailed below). The differential rope skipping group (DG), n = 13, was on average 13.8 ± 0.7 years old; the control group (CG), n = 13, was 14.0 ± 0.7 years old. In both groups, the ratio between male and female (7 female and 6 male each) as well as competitive athletes (1:1) was balanced. The study was conducted according to the guidelines of the Declaration of Helsinki and approved by the Ethics Committee of the Johannes Gutenberg-University Mainz (2021/10; 25 October 2021). Furthermore, the parents as well as the students were informed and gave informed consent before the study.

### 2.2. Design

Rope skipping, as a motor activity, was chosen on the one hand because it combines cardio-vascular and coordination aspects and on the other hand because it can be performed in a spatially limited way on the school grounds (break yard/auditorium/corridors). In addition, the studies by John and Schöllhorn [11] and John et al. [30] provided interesting insights into the relationship between cardiac and brain activity and training method during rope skipping. Another reason are approaches from the research of Brümmer et al. [2], who found that there are greater cortical adaptations (more favorable brain states for learning) during running than cycling or swimming, which could be due to the mechanical vibration generated by the rhythmic ground contact [11,30,73].

**Figure 1:**
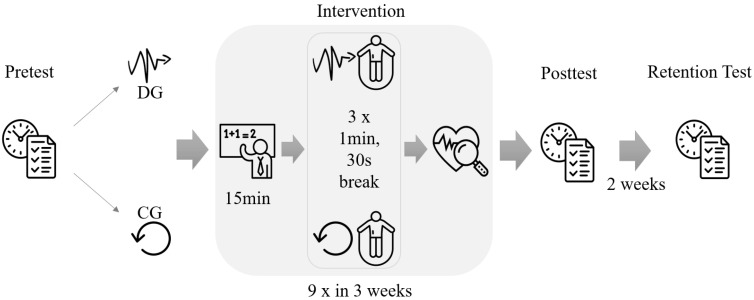
Schematic study procedure with time schedule. The intervention procedure outlined was conducted a total of 9 times over a three-week period. DG = differential rope skipping group; CG = control group.

Figure 1 shows schematically the procedure of the five-week study with a pre-, post, and retention test. All testing and the interventions were conducted during regular school hours. First, a pretest was conducted, based on which the participants were alternately assigned to the DG and the CG according to descending total score. This was followed by a three-week intervention period with 9 sessions conducted on Mondays, Wednesdays, and Fridays at 9 AM during their second hour of school. According to Maynard and colleagues [74], the intervention period and number of sessions should be sufficient to find possible cognitive effects. The duration of intervention and number of sessions was oriented on previous studies on DL [75,76]. Additionally, it was aligned with the school’s curriculum, which typically calls for exams after the fourth to sixth week of a learning module, and we wanted to avoid clashing with an exam period due to the increased stress level of the participants. Each intervention session was structured in the same way: First, 15 min of math was taught. This was followed by 3 times 1 min of rope skipping with two 30-s breaks to coarsely control the intensity of the metabolism, measured by the heart rate. After rope skipping, participants immediately determined their own heart rate by hand for load monitoring. This procedure was practiced intensively with the participants in advance. After the three-week intervention period, the posttest was performed, and after another two weeks without intermediate intervention (e.g., exam), the retention test was performed. The same experimenter conducted all the testing, math teaching, and rope skipping sessions. All students participated in at least eight of the nine math learning sessions and performed all tests.

Participants in the CG were instructed to jump as consistently as possible in a steady rhythm at a constant speed, according to the repetitive learning approach [69]. The jumping rhythm, whether with or without intermediate jumps, as well as the rhythm frequency could be chosen individually, but should feel comfortable due to the intensity control. The DG performed jumping according to the criteria of DL [21,22]; an overview of all exercises is shown in Table A1. During the 30 s breaks and after every 20 s of rope skipping, new movement instructions were given to the students. This occurred regardless of the quality of execution of the previous task. The resulting task frequency of 3 tasks per minute attempted to avoid stressful overload with too many new tasks [11]. In case of failure in jumping, students were asked by the experimenter simply to continue. Neither emotional nor augmented feedback on execution or corrections were given.

The contents of the 15 min math session were exercises and solution strategies for mental arithmetic and fractions; the specific topics of each math session are listed in Table A1.

The written cognitive test in math consisted of arithmetic problems in mental arithmetic and fractions, which are considered to support the understanding of the magnitude of numbers [77]. The understanding of the size of fractions is considered to play an important role in general mathematics performance [78].

**Figure A1:**
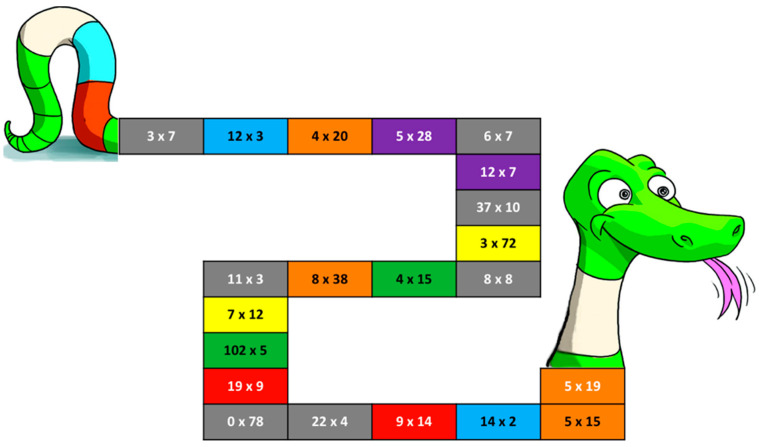
Exemplary version of the mental arithmetic test. The mental arithmetic part consisted of 21 tasks that had to be solved under time pressure. Every three seconds a new task appeared, each of which was shown for 9 s. Consequently, a maximum of three tasks were shown simultaneously and the resulting duration of the mental arithmetic part was 61 s. The tasks for the posttest and retention test were slightly modified from the pretest without changing the difficulty of the tasks and the task structure.

The mental arithmetic part (Figure A1) consisted of 21 tasks that had to be solved under time pressure. Every three seconds a new task appeared on the screen, each of which was shown for 9 s. Consequently, a maximum of three tasks were shown simultaneously and the resulting duration of the mental arithmetic part was 61 s. For example, tasks like 12∗3, 3∗72 and 8∗38 had to be solved.

**Figure A2:**
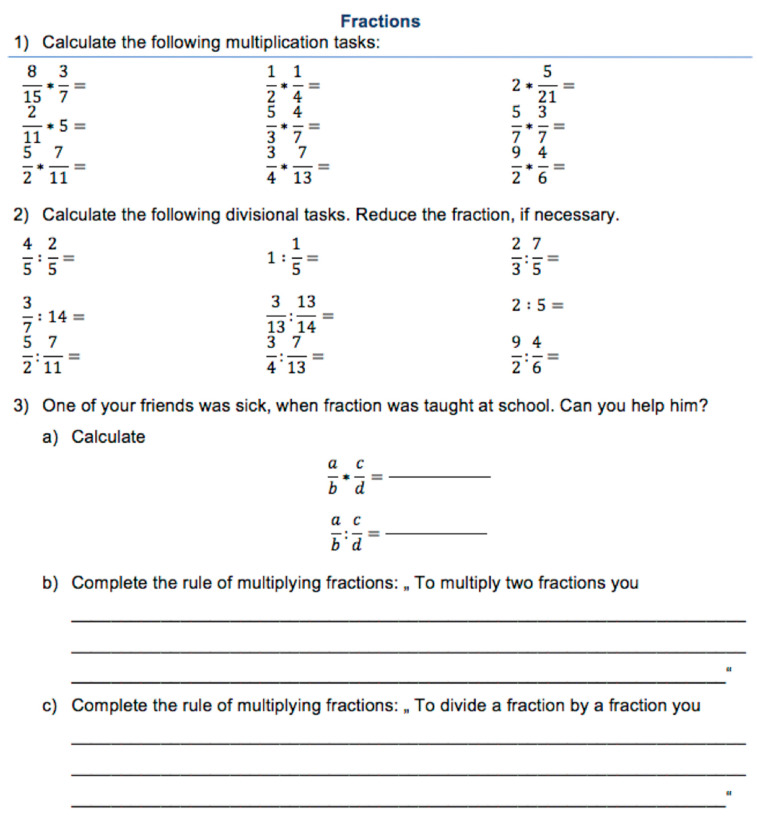
Exemplary version of the fractions test. The part in the arithmetic test about fractions consisted of 21 tasks: nine tasks concerned multiplying fractions, nine tasks dividing fractions and three tasks formulating general rules. The test persons had five minutes time for all tasks. The tasks for the posttest and retention test were slightly modified from the pretest without changing the difficulty of the tasks and the task structure.

The part in the arithmetic test, (Figure A2), about fractions consisted of 21 tasks: nine tasks concerned multiplying fractions, nine tasks dividing fractions and three tasks formulating general rules. The test persons had five minutes for all tasks. Exemplary, tasks were: $\frac{6}{15}*\frac{3}{7}$, $\frac{3}{5}:\frac{7}{13}$ or $\frac{a}{b}:\frac{c}{d}$.

All students performed the entire test at the simultaneous time. They started with the mental arithmetic part and after this was finished, the fractional arithmetic part was performed. The tasks for the post- and retention test were slightly modified from the pretest without changing the difficulty of the tasks and the task structure (e.g., 12∗3 became 13∗3 or $\frac{6}{15}*\frac{3}{7}$ became $\frac{9}{15}*\frac{5}{7}$).

### 2.3. Statistical Analyses

To estimate the optimum sample size, a statistical power analysis with GPower [79], based on Neyman–Pearson statistics [80], was performed, using data from studies by Henz et al. [27,28,29], comparing repetitive with DL. The effect size in these studies was *η^2^* = 0.31 (n = 24), *η^2^* = 0.16 (n = 12), *η^2^* = 0.17 (n = 22), which were considered large to extremely large. With an *α* = 0.05 and a power of 0.80, the projected sample size required for these effect sizes is approximately between n = 8 and n = 26 for this simplest intergroup comparison. The sample size of n = 26 proposed by us is therefore sufficient for the main objective of this study and should also consider the expected fluctuation and our additional objectives of controlling possible subgroup analyses.

The groups were compared based on their scores on the mental arithmetic and fractions subtests and on the total test. For the total test results, the partial results of the mental arithmetic and fractions tests were summed with equal weighting.

Analysis of the data using the Shapiro–Wilk test revealed that some variables violated the criterion of normal distribution. Therefore, nonparametric statistical tests were used to analyze the development of the groups across the measurement time points and to compare the groups at each measurement time point.

For the analysis of the development within groups from pre- through post- to retention test, the results of the tests were statistically compared using Friedman test. In case of significant results, pairwise Bonferroni-corrected Dunn–Bonferroni post hoc tests were performed.

To compare the score between the groups at pre-, post-, and retention test, test results were statistically compared using Mann–Whitney U Tests. The comparison at the time of the pretest also represents the basis of the test for homogeneity.

A statistical comparison of the heart rate between DG and the CG after rope skipping was calculated by Mann–Whitney U Tests.

In addition, the effect size *r* was calculated for the pairwise post hoc tests of the Friedman as well as for the Mann–Whitney U Tests. Here, 0.1 ≤ *r* < 0.3 corresponds to a weak effect, 0.3 ≤ *r* < 0.5 to a medium effect, and *r* ≥ 0.5 to a strong effect [81].

The *p* -value at which it is considered worthwhile to continue research according to the original Fisher statistics [82] was set at *p* = 0.05.

## 3. Results

**Figure 2:**
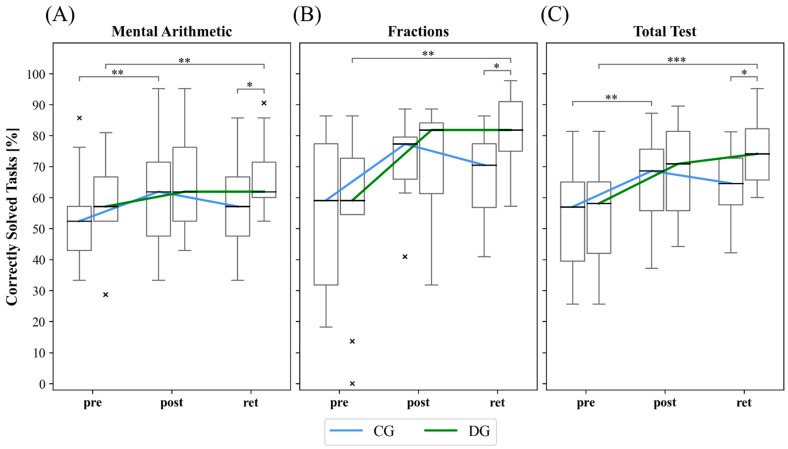
Development of the groups in the tests over the duration of the examination. Values are considered as outliers if they are outside the interval [Q1 − 1.5 * (Q3 − Q1), Q3 + 1.5 * (Q3 − Q1)]. × = outlier (each × stands for one outlier). Brackets show pairwise significant differences (* *p* ≤ 0.05; ** *p* ≤ 0.01; *** *p* ≤ 0.001). Shown are boxplots of the mental arithmetic test (**A**), the fractions test (**B**) and the total test (**C**). For the clarity of the development of the groups, the median curves are also shown by line plots. CG = control group; DG = differential learning group; Pre = pretest; Post = posttest; Ret = retention test.

The math test results of the DG and CG on each test are shown in Figure 2A–C. The results of the statistical analyses of the development within the groups, the comparison between the groups, and the comparison of the heart rate between the groups are shown in the following subsections, respectively.

### 3.1. Development within Groups over Measurement Time Points

As Table 2 shows, the group that did differential rope skipping after the math lessons improved their math performance statistically significant over the course of the study in both subtests and in the total test (*p* ≤ 0.006). In each of the mental arithmetic, fractions, and total tests, there was a statistically significant improvement with a medium effect size over the course from pre- to retention test (*p* ≤ 0.01, *r* ≥ 0.320). In the acquisition phase, there are only statistically significant trends with weak effect sizes (0.056 ≤ *p* ≤ 0.118, 0.224 ≤ *r* ≤ 0.256). In the retention phase, there was no development in the mental arithmetic test, but in the fractions test and in the total test, trends with weak effect size can be observed (0.056 ≤ *p* ≤ 0.093, 0.235 ≤ *r* ≤ 0.256).

The CG improved statistically significant over the course of the study in mental arithmetic and the total test (*p* ≤ 0.003). There was no statistically significant change in the fractions test. In the acquisition phase, significant improvements were shown in mental arithmetic and in the total test, each with a medium effect size (*p* ≤ 0.005, 0.341 ≤ *r* ≤ 0.363). In the retention phase, there was no further statistical change. Over the course from pre- to retention test, a statistical trend with weak effect size can be observed in the total test (*p* = 0.072, *r* = 0.235).

### 3.2. Comparison between Groups across Measurement Time Points

As presented in Table 3, testing the homogeneity of DG and CG in the pretest with the Mann-Whitney U test revealed no statistically significant differences in all arithmetic tests (*p* ≥ 0.390). There were also no statistically significant differences between the groups on any of the tests at posttest. In the retention test for both the mental arithmetic and fractions subtests and the overall test, the DG scored significantly better in each case with a medium effect size (*p* ≤ 0.039, *r* ≥ 0.407).

**Figure 3:**
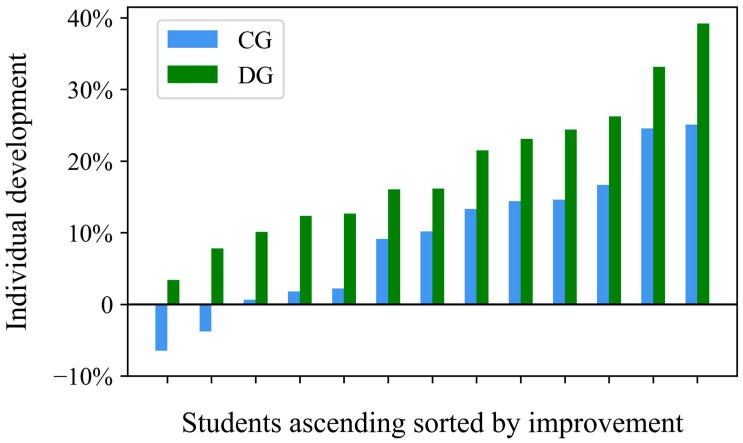
Individual development of each student of both groups over the entire study period in the performance of the total test sorted in ascending order and compared in pairs. The *x*-axis shows the DG and CG students in pairs, after they have been ranked within each group according to learning progress. The *y*-axis shows the difference in percentage points between pretest and retention test. CG = control group; DG = differential learning group.

Looking at the individual development over the course of the study (Figure 3), we see that all students in the DG improved from the baseline level, while in the CG even two students worsened, and three others remained at about the baseline level and improved only marginally.

Furthermore, comparing the students individually with the respective best, second-best, third best, etc. development in the respective groups, it is noticeable that in all pairwise comparisons the student of the DG performed better. The fourth best of the DG improved in the same way as the best of the CG and the worst of the DG improved even more than the fifth worst of the CG.

### 3.3. Comparison of Heart Rates between the Groups

When comparing heart rates (Table 4) no statistically significant difference can be identified between groups on average across all sessions (*p* = 0.880, *r* = 0.030). Looking more closely at the individual sessions, significantly higher heart rates can be seen in the CG for the first (*p* = 0.002, *r* = 0.599) and second (*p* = 0.039, *r* = 0.403) sessions. There was no statistically significant difference between CG and DG for the other seven units.

## 4. Discussion

In this study, we investigated the influence of rope skipping according to the DL approach [21,22], on cognitive improvement in the area of mathematics learning in fractions and mental arithmetic. A rope skipping group according to the motor learning approach of repetition learning [69] served as a control group. We found indications for a sustainable impact of sport activities following the math lessons on learning performance for the math acquisition. In the acquisition phase between pretest and posttest, the mathematical skills of the DG and CG developed to a similar extent. In contrast, in the subsequent learning phase following the post- up to the retention test, however, the performance of the DG increased further, while that of the repetitive group decreased somewhat following a typical memory-forgetting curve. This tendency affected both the mental arithmetic ability and fractional arithmetic ability tests and, consequently, development across the total test. Thus, the results could also provide indirect indications for cognitive constructs underlying mathematics learning, such as working memory or executive functions, although this would be subject to future research. Particularly interesting are the qualitative comparisons at the individual level, where each participant of the DG achieved a greater learning progress in the mathematics test compared to pendant of the CG. In addition, all subjects in the DG achieved positive learning progress, which was not the case for the CG.

In general, numerous studies have shown that physical exercise can act as cognitive enhancers both acutely and in the long term [31,32,33,39,40,41,42,43,44,83]. However, two aspects have been neglected in research so far. First, the chronological order of physical exercise and cognitive learning, and second, the effects of different types of physical exercise and the diversity therein on actual learning performance. This study provides indications that cognitive learning could be positively influenced by subsequent physical activity. In addition, the type of execution also seems to play a major role. Highly variable physical exercises according to the DL approach led to significantly better results in learning progress than the repetitive execution of physical exercise.

The repeated and continuous increase in performance of the DG after completion of the acquisition phase could be an indication of medium-term effects, efficiency, and sustainability of learning. In addition, the exercise interventions, which lasted only three times a minute after each session, were significantly shorter and metabolism wise less intense than previous studies of the relationship between exercise and cognition, which lasted at least 20 min [2,3,5,6,33,34] or required maximal intensity [4,6,19]. This would be of great advantage for everyday school life, as such learning support could be carried out several times a day without excessive additional space and costs. Sports learning support based on endurance or high intensity sports, for example, could only be realized with a significantly lower frequency on one day and during the week due to the duration and load and the corresponding necessary recovery time.

### 4.1. Explanation Models for Improved Learning Progress

To gain possible mechanisms of action and explanatory approaches, we first look at possible metabolic mechanism caused by the intensity with which both groups exercised. Measuring the intensity of rope skipping based on heart rate showed no statistical difference between the DG and CG. Only in the first and second training session the intensity was slightly higher in the CG. A possible reason for the difference could be that the subjects were not used to the differential rope skipping, so they might have expected possible feedback after getting stuck on the rope and waited and did not continue directly. This may have led to a reduction in movement time and thus intensity. Since overall, no significant difference in heart rate was measured between the DG and the CG, cardiovascular data do not support the further pursuing of this question according to Fisher’s interpretation of statistics. The results of the study provide indications that the cardiovascular load during rope skipping and an associated, e.g., increased blood flow to the brain can hardly be regarded as the cause for the different performance improvement of the DG and the CG in arithmetic. While the most obvious metabolic mechanisms do not seem to be sufficient to explain the results, BDNF may play a more important role. Studies on BDNF levels in adolescents suggest that this can be increased by aerobic activity [65,66]. However, it remains open whether the type of exercise may also play an important role here and could lead for a faster BDNF increase [84]. Whether the better results of the DG can be explained by an increased BDNF level should be specifically investigated in future studies.

Another approach to frame the results is provided by studies on retroactive interference. According to models for memorization, attention-intensive tasks impair the consolidation of previously learned information by inhibiting an active process of memory activation and consolidation that would otherwise occur at that time [85,86,87,88,89,90,91]. However, specific features of task structure that promote or inhibit memory consolidation are yet only known to a limited extent. Collins and Wamsley [92] found that tasks that directed focus away from memory processes led to better memory consolidation than tasks associated with internal focus, as retrieval of memories would often be devoted to internal focus. However, memory-driven cognitive processes would activate the hippocampus [93,94], which in turn could block memory consolidation [86]. Collins and Wamsley [92] concluded that either absolute rest in the form of deep meditation or tasks with low breath focus would be helpful for memory consolidation. The results in this study could provide yet another starting point by attempting to completely tax the attentional resources through movement and thus completely prevent them from detrimental memory effects. The coordinatively highly demanding movement performance in DL may generate brain activities that indicate somatosensory processes of working memory, which in turn provide particularly high resources for information processing. As a result, the memory trace becomes more stable in the face of internal and external disturbances related to executive control processes such as attentional processes [28] and the memory consolidation of what has been cognitively learned could have been positively influenced afterwards. 

A further approach to explain the results of the study and partially underpin the previous logic of interpretation could lie in neurophysiological mechanisms. According to the hypo-frontality-hypothesis [34], the reduction in activity in the frontal and pre-frontal brain regions in the form of increased alpha and theta activation would increase the likelihood of reaching a brain state conducive to learning [68]. Previous studies considering the relationship between brain activity and sport motor training methods [26,27,29] found increased frontal and central theta activity as well as increased central and posterior alpha activity as a specific feature of training according to the DL approach. John and Schöllhorn [11] also found differences in brain activity between repetitive and differential rope skipping and even between the amount of noise within DL [30]. However, the extent to which brain activity through DL directly influences previous cognitive learning would need to be specifically investigated in follow-up studies. The same is to the specific vertical movements and its mechanical stimulation [73].

In analogy to [11], the extent to which the observed effects can be attributed to the cognitive processes involved in creating new rope-skipping variants or only by executing them requires future research. 

Another approach to view the results in a different context could be provided by learning research in the context of sleep. It is not doubtful that sleeping or napping after learning can enhance the effect of cognitive learning [56,57,58,59]. Henz and Schöllhorn [95] found that DL produced similar cortical frequency bands in cortical areas relevant to learning as during NREM sleep or meditation, although not to the same extent. By producing similar brain waves thru vertical hopping similar mechanisms could have been initiated. However, the relationship between DL and sleep requires future intensive research before conclusions can be drawn in this context.

### 4.2. Limitations

The entire investigation was conducted during regular math lessons at school with higher ecological validity, which led to some methodical limitations. Due to limited equipment, possibilities and time conditions, the tested students had to determine their heart rate themselves. However, this procedure was trained beforehand, which is no guarantee that the individual heart rates could be reliably determined. In addition, it always took a few seconds before each measurement for all participants to detect their pulse, which could have led to a slight drop of the heart rate. Nonetheless, one could argue that the measured mean heart rate suggests a relatively low intensity. However, studies also showed that cognitive learning can be positively influenced during physical activity under low intensity [96,97]. Overall, the determined heart rates provide indications that the metabolic load of the two groups was almost the same. Furthermore, the basic resting heart rate as well as external circumstances or mood could not be re-examined before each learning unit due to the lack of time. This, however, depicts everyday school life and puts the results into a realistic context. It should be kept in mind that this is only a first and a purely phenomenological study in which different possible explanations for the different learning rates of both groups are presented. An EEG measurement of the test persons might lead to more detailed findings in the future.

## 5. Conclusions

A positive influence of sport and physical activity on cognitive performance has been demonstrated many times. However, the aspect of precise timing of the sport activity relative to the learning unit, with additional consideration of the specifics of selected motor learning approaches, has been largely neglected. The present study provided evidence for the relevance of motor activities immediately following cognitive learning sessions. In this context, the type of physical activity seems to depend on the underlying learning approach. Looking at the groups, math performance improved better by means of rope skipping in the type of the DL than the repetition-oriented approach. This is consistent with brain activations found in previous studies that showed advantages for learning and consolidation processes in DL [28,98]. A key element here seems to be the simultaneous combination of coordinatively demanding and whole-body movements with metabolic activating characteristics. Future research will show to what extent the effects can be enhanced by successive combinations of the two elements or by individually optimized alternating frequencies in rope hopping [11,30]. Whether the adding of music [99] for the initiation of certain emotions [100] or changing the number of skipping series to achieve different grades of fatigue [101] will change the effectivity to the better or to the worse also needs further research. Especially under the consideration of inconvenient and unexpected side effects of biochemical or other alternatives for cognitive enhancement and under consideration of the original interpretation of Fisher statistics [82], the results show that it is worthwhile to continue research in this direction.

## Figures and Tables

**Table 1:** Structural overview of studies that directly temporally relate movement to cortical activity or cognitive learning performance.

Study	Type of Exercise	Structure of Exercise Sequence			Type of Test		Amount (Intensity)		Relative Moment	
		Blocked	Serial/Random	Differential	Indirect	Direct	Duration (Intensity)	Frequency	Before	After
[2]	Cardio	x			x		30 min	1x	x	
[15]	Coord	x				x	10 min	1x	x	
[17]	Strength	x				x	20 min (70 10RM)	2x	x	
[18]	Strength	x				x	20 min (70% 10RM)	2x	x	
[7]	Cardio	x				x	30 min (moderate)	1x	x	
[26]	Coord	x		x	x		15 min	1x	x	
[29]	Coord	x		x	x		15 min	1x	x	
[27]	Coord	x	x	x	x		15 min	1x	x	
[28]	Coord	x	x	x	x		15 min	1x	x	
[9]	Cardio	x				x	20 min (60%)	1x	x	
[10]	Coord	x				x	15 min	1x	x	
[11]	Coord	x		x	x		1 min	1x	x	
[30]	Coord	x		x	x		1 min	1x	x	
[8]	Cardio	x				x	30 min (40%)	1x	x	
[23] ^R^	Coord	x	x		x	x	15–30 min	1x	x	
[12] ^R^	Cardio	x				x	10–100 min (Inverted-U, Steady state, Fatigue)	1x	x	
[16]	Cardio	x				x	30 min (60–70%)	1x	x	
	Strength						30 min (80% 1RM)			
[5]	Cardio	x			x		21–60 min		x	
[4]	Cardio	x			x		~15 min (100%)	1x	x	
[6] ^R^	Cardio	x				x	20–60 min (moderate to maximal)	1x	x	
[19]	Sprint	x			x		6 min (maximum)	1x	x	

^R^: review article; Cardio = Cardiovascular activity; Coord = Coordinative activity; Strength = Strength related activity; Sprint = sprint running. Indirect type of test contains EEG, fMRI, dMRI, or Blood analyses; direct type of tests contain tests related to cognitive functions such as reaction time, movement time, response time, attention, executive functions, mental rotation, perception speed and accuracy. The table does not claim to be complete.

**Table 2:** Statistical comparisons at the three measurement time points within groups.

Comparison	Friedman Test (Rank Scores)	Post Hoc Dunn–Bonferroni Tests
Mental Arithmetic		
CG: Pre–Post–Ret	*χ*2(2) = 11.636, *p* = 0.003 ** (Pre: 1.38; Post: 2.62; Ret: 2.00)	Pre vs. Post: *p* = 0.005 **, *r* = 0.341 ^++^ Pre vs. Ret: *p* = 0.350, *r* = 0.171 ^+^ Post vs. Ret: *p* = 0.350, *r* = 0.171 ^+^
DG: Pre–Post–Ret	*χ*2(2) = 10.085, *p* = 0.006 ** (Pre: 1.35; Post: 2.15; Ret: 2.50)	Pre vs. Post: *p* = 0.118, *r* = 0.224 ^+^ Pre vs. Ret: *p* = 0.01 **, *r* = 0.320 ^++^ Post vs. Ret: *p* = 1.000, *r* = 0.096
Fractions		
CG: Pre–Post–Ret	*χ*2(2) = 4.545, *p* = 0.103 (Pre: 1.62; Post: 2.38; Ret: 2.00)	–
DG: Pre–Post–Ret	*χ*2(2) = 18.615, *p* < 0.001 *** (Pre: 1.15; Post: 2.00; Ret: 2.85)	Pre vs. Post: *p* = 0.093, *r* = 0.235 ^+^ Pre vs. Ret: *p* < 0.001 ***, *r* = 0.469 ^++^ Post vs. Ret: *p* = 0.093, *r* = 0.235 ^+^
Total Test		
CG: Pre–Post–Ret	*χ*2(2) = 11.804, *p* = 0.003 ** (Pre: 1.27; Post: 2.58; Ret: 2.15)	Pre vs. Post: *p* = 0.003 **, *r* = 0.363 ^++^ Pre vs. Ret: *p* = 0.072, *r* = 0.245 ^+^ Post vs. Ret: *p* = 0.842, *r* = 0.117 ^+^
DG: Pre–Post–Ret	*χ*2(2) = 22.154, *p* < 0.001 *** (Pre: 1.08; Post: 2.00; Ret: 2.92)	Pre vs. Post: *p* = 0.056, *r* = 0.256 ^+^ Pre vs. Ret: *p* = 0.001 ***, *r* = 0.512 ^+++^ Post vs. Ret: *p* = 0.056, *r* = 0.256 ^+^

Note. All *p*-values of the post hoc tests are Bonferroni-corrected. CG = control group; DG = differential learning group; Pre = pretest; Post = posttest; Ret = retention test. ** *p* ≤ 0.01; *** *p* ≤ 0.001; **^+^** 0.1 ≤ *r* < 0.3; **^++^** 0.3 ≤ *r* < 0.5; **^+++^** *r* ≥ 0.5.

**Table 3:** Statistical comparisons at the three measurement time points between groups.

Measurement Time Point	Group	Mdn	RS	Mann–Whitney-U
Mental Arithmetic				
Pre	CG	0.571	12.15	*U* = 67, *p* = 0.390, *r* = 0.176 ^+^
	DG	0.571	14.85	
Post	CG	0.619	12.77	*U* = 75, *p* = 0.650, *r* = 0.096
	DG	0.619	14.23	
Ret	CG	0.610	10.31	*U* = 134, *p* = 0.034 *, *r* = 0.421 ^++^
	DG	0.619	16.69	
Fractions				
Pre	CG	0.590	13.65	*U* = 86.5, *p* = 0.920, *r* = 0.020
	DG	0.590	13.35	
Post	CG	0.773	12.96	*U* = 77.5, *p* = 0.724, *r* = 0.071
	DG	0.818	14.04	
Ret	CG	0.764	10.12	*U* = 131.5, *p* = 0.022 *, *r* = 0.443 ^++^
	DG	0.818	16.88	
Total Test				
Pre	CG	0.581	14.00	*U* = 169, *p* = 0.762, *r* = 0.066
	DG	0.576	13.00	
Post	CG	0.698	14.04	*U* = 168.5, *p* = 0.724, *r* = 0.070
	DG	0.709	12.96	
Ret	CG	0.704	16.62	*U* = 135, *p* = 0.039 *, *r* = 407 ^++^
	DG	0.741	10.38	

Note. Mdn = Median; RS = Rank Scores; CG = control group; DG = differential learning group; Pre = pretest; Post = posttest; Ret = retention test. * *p* ≤.05; **^+^** 0.1 ≤ *r* < 0.3; **^++^** 0.3 ≤ *r* < 0.5.

**Table 4:** Statistical comparisons of the heart rates over the learning sessions between the groups.

Session		1		2		3		4		5		6		7		8		9		Mean	
		Mdn	RS	Mdn	RS	Mdn	RS	Mdn	RS	Mdn	RS	Mdn	RS	Mdn	RS	Mdn	RS	Mdn	RS	Mdn	RS
CG		120	18.08	120	16.58	106	12.85	110	16.12	96	11.62	110	11.77	100	14.27	94	12.38	102	14.31	109	13.27
DG		88	8.92	99	10.42	119	14.15	98	10.88	108	15.38	114	15.23	98	12.73	115	14.62	100	12.69	100	13.73
Mann–Whitney U-Test	*U*	144		124.5		67		118.5		60		62		94.5		70		95		81.5	
	*p*	0.002 **		0.039 *		0.687		0.081		0.223		0.246		0.614		0.479		0.614		0.880	
	*r*	0.599 ^+++^		0.403 ^++^		0.086		0.342 ^++^		0.247 ^+^		0.228 ^+^		0.101 ^+^		0.146 ^+^		0.106 ^+^		0.030	

Note. Mean of each heart rate of each subject. Mdn = Median; RS = Rank Scores; CG = control group; DG = differential learning group. * *p* ≤ 0.05; ** *p* ≤ 0.01; **^+^** 0.1 ≤ *r* < 0.3; **^++^** 0.3 ≤ *r* < 0.5; **^+++^** *r* ≥ 0.5.

## Data Availability

The data that support the findings of this study are available from the corresponding author, J.B., upon reasonable request.

## Appendix A

**Table A1:** Content the math sessions and the rope skipping task of the DG. The task of the CG was to jump as consistently as possible in a steady rhythm at a constant speed. The jumping rhythm, whether with or without intermediate jumps, as well as the speed could be chosen individually.

Session	Cognitive Task (15 min)	Tasks for Differential Rope Skipping Group (3 × 1 min; 30 s Break)
1	Mental arithmetic: Neighbor tasks e.g., 20∗9=20∗10−20	Rhythm: 20 s 2 hops/1 swing; 20 s 1 hop/1 swing; 20 s 1 hop/2 swings Landing: 20 s toes, 20 s heel, 20 s alternating Feet different: one foot landing on toes the other one on the heel: 20 s circles of 3 jumps; 20 s circles of 2 jumps; 20 s alternating
2	Mental arithmetic Change Order: e.g., 14∗2=2∗14	Free choice of hops per swing ratio. Foot position: 30 s toes pointing towards, 30 s toes pointing opposite Foot position: 30 s toes point to the left, 30 s toes point to the right Foot position: 5× left, 4× right, 3× left, 2× right, … alternating; afterwards rising again
3	Mental arithmetic: Factor decomposition: e.g., 7∗12=7∗10+7∗2	Swinging backwards; Rhythm: 20 s 2 hops/1 swing; 20 s 1 hop/1 swing; 20 s 1 hop/2 swings 30 s max. frequency; 30 s max. jumping height Backwards; 30 s max. frequency; 30 s max. jumping height
4	Mental arithmetic: Halve/double: e.g., $5*18=\frac{10*18}{2}$	Analogous to Session 2, however jumping backwards
5	Mental arithmetic: Mixed training of the previously learned mental arithmetic techniques.	Free choice of hops per swing ratio. 1 min: Leg position: 4× crossed, left in front; 2× parallel, 4× crossed right in front; 2× parallel (continuously) 1 min: Legs crossed. Switch leg in top on command (varying intervals) 1 min: Same task, but backwards
6	Fractions: General introduction of multiplying and dividing fractions	Rhythm: 2 hops/1 swing Landing on hop between swings: 20 s legs in straddle position; 20 s lunge, left foot in front; 20 s lunge, right foot in front 1 min: analogous: circles of 5, of 4,… alternating 1 min: analogous, but backwards
7	Fractions: Consolidation of multiplying fractions	Jumping while walking on the spot 20 s normal; 20 s knees stretched; 20 s highly raised knees 20 s high knee skips; 20 s bring heel to the bottom; 20 s max. height: high knee skips 1 min: 5× knees stretched, 5× highly raised knees 5× high knee skips, 5× heel at bottom; all 4×; 3×,… alternating
8	Fractions: Consolidation of dividing fractions	Rope position: 15 s swinging besides the body on the right side; 15 s left side; 30 s 2 normal jumps 1 jump swinging the rope besides the body 30 s 2 jumps, rope swing left; 30 s 2 jumps, rope swing right. 1 min: Jumping: on command 1: rope swing right; command 2 rope swing left (varying intervals)
9	Fractions: Mixed training of multiplying and dividing fractions	While running: 30 s 2 jumps, rope swing left, 2 jumps, rope swing right; 30 s 3 jumps, rope swing left, rope swing right 1 min Analogous with stretched knees 1 min: high knee skips: On command 1: rope swing right; command 2: rope swing left; command 3: rope swings left and right; command 4 rope swings right and left
